# Yoga for asthma

**DOI:** 10.1590/1516-3180.20161344T2

**Published:** 2015-11-13

**Authors:** Zu-Yao Yang, Hui-Bin Zhong, Chen Mao, Jin-Qiu Yuan, Ya-Fang Huang, Xin-Yin Wu, Yuan-Mei Gao, Jin-Ling Tang

## Abstract

**BACKGROUND:**

Asthma is a common chronic inflammatory disorder affecting about 300 million people worldwide. As a holistic therapy, yoga has the potential to relieve both the physical and psychological suffering of people with asthma, and its popularity has expanded globally. A number of clinical trials have been carried out to evaluate the effects of yoga practice, with inconsistent results.

**OBJECTIVES:**

To assess the effects of yoga in people with asthma.

**METHODS:**

*Search methods:* We systematically searched the Cochrane Airways Group Register of Trials, which is derived from systematic searches of bibliographic databases including the Cochrane Central Register of Controlled Trials (CENTRAL), MEDLINE, EMBASE, CINAHL, AMED, and PsycINFO, and handsearching of respiratory journals and meeting abstracts. We also searched PEDro. We searched ClinicalTrials.gov and the WHO ICTRP search portal. We searched all databases from their inception to 22 July 2015, and used no restriction on language of publication. We checked the reference lists of eligible studies and relevant review articles for additional studies. We attempted to contact investigators of eligible studies and experts in the field to learn of other published and unpublished studies.

*Selection criteria:* We included randomized controlled trials (RCTs) that compared yoga with usual care (or no intervention) or sham intervention in people with asthma and reported at least one of the following outcomes: quality of life, asthma symptom score, asthma control, lung function measures, asthma medication usage, and adverse events.

*Data collection and analysis:* We extracted bibliographic information, characteristics of participants, characteristics of interventions and controls, characteristics of methodology, and results for the outcomes of our interest from eligible studies. For continuous outcomes, we used mean difference (MD) with 95% confidence interval (CI) to denote the treatment effects, if the outcomes were measured by the same scale across studies. Alternatively, if the outcomes were measured by different scales across studies, we used standardized mean difference (SMD) with 95% CI. For dichotomous outcomes, we used risk ratio (RR) with 95% CI to measure the treatment effects. We performed meta-analysis with Review Manager 5.3. We used the fixed-effect model to pool the data, unless there was substantial heterogeneity among studies, in which case we used the random-effects model instead. For outcomes inappropriate or impossible to pool quantitatively, we conducted a descriptive analysis and summarized the findings narratively.

**MAIN RESULTS:**

We included 15 RCTs with a total of 1048 participants. Most of the trials were conducted in India, followed by Europe and the United States. The majority of participants were adults of both sexes with mild to moderate asthma for six months to more than 23 years. Five studies included yoga breathing alone, while the other studies assessed yoga interventions that included breathing, posture, and meditation. Interventions lasted from two weeks to 54 months, for no more than six months in the majority of studies. The risk of bias was low across all domains in one study and unclear or high in at least one domain for the remainder.

There was some evidence that yoga may improve quality of life (MD in Asthma Quality of Life Questionnaire (AQLQ) score per item 0.57 units on a 7-point scale, 95% CI 0.37 to 0.77; 5 studies; 375 participants), improve symptoms (SMD 0.37, 95% CI 0.09 to 0.65; 3 studies; 243 participants), and reduce medication usage (RR 5.35, 95% CI 1.29 to 22.11; 2 studies) in people with asthma. The MD for AQLQ score exceeded the minimal clinically important difference (MCID) of 0.5, but whether the mean changes exceeded the MCID for asthma symptoms is uncertain due to the lack of an established MCID in the severity scores used in the included studies. The effects of yoga on change from baseline forced expiratory volume in one second (MD 0.04 liters, 95% CI -0.10 to 0.19; 7 studies; 340 participants; I^2^ = 68%) were not statistically significant. Two studies indicated improved asthma control, but due to very significant heterogeneity (I^2^ = 98%) we did not pool data. No serious adverse events associated with yoga were reported, but the data on this outcome was limited.

**AUTHORS CONCLUSIONS:**

We found moderate-quality evidence that yoga probably leads to small improvements in quality of life and symptoms in people with asthma. There is more uncertainty about potential adverse effects of yoga and its impact on lung function and medication usage. RCTs with a large sample size and high methodological and reporting quality are needed to confirm the effects of yoga for asthma

The full text of this review is available from: http://onlinelibrary.wiley.com/doi/10.1002/14651858.CD010346.pub2/full
